# Gemcitabine inhibits immune escape of pancreatic cancer by down regulating the soluble ULBP2 protein

**DOI:** 10.18632/oncotarget.11780

**Published:** 2016-09-01

**Authors:** Xiansheng Lin, Mei Huang, Fang Xie, Hangcheng Zhou, Ji Yang, Qiang Huang

**Affiliations:** ^1^ Department of General Surgery, Affiliated Provincial Hospital of Anhui Medical University, Hefei, 230001, China; ^2^ Anhui Province Key Laboratory of Hepatopancreatobiliary Surgery, Hefei, 230001, China; ^3^ Department of Pathology, Affiliated Provincial Hospital of Anhui Medical University, Hefei, 230001, China

**Keywords:** pancreatic cancer, gemcitabine, ULBP2, ADAM10, NK cells

## Abstract

Due to early onset of local invasion and distant metastasis, pancreatic cancer is the most lethal human malignant tumor, with a 5 year survival rate of less than 5%. As a effective chemotherapy drug for pancreatic cancer patients, gemcitabine is reported to inhibit cell proliferation as a nucleotide analog. In this study, we investigated the role of gemcitabine in immune regulation of pancreatic cancer. Our data showed that the level of soluble ULBP2 (sULBP2), a ligand of NKG2D receptor, decreased in the supernatants of pancreatic cancer cell lines when gemcitabine was added, and sULBP2 level correlated with NK92 cells cytotoxicity to pancreatic cancer cell lines. Importantly, our data showed that gemcitabine promoted PANC-1 cells and MIA PaCa-2 immune evasion by reducing ADAM10 expression, a metalloproteinase involved in sULBP2 shedding from cell membrane. Knockdown of ADAM10 clearly downregulated sULBP2 levels in the culture supernatants and cells became more susceptible to NK92 cytotoxicity. Serum samples and tumor samples were obtained from 45 patients with pancreatic ductal adenocarcinoma (PDAC). Statistical analysis showed a significant correlation between the serum level of sULBP2 with ADAM10 expression in PDAC tissues. In conclusion, our data demostrated that gemcitabine inhibits ULBP2 ectodomain shedding through the suppression of ADAM10 and enhance NK cells cytotoxicity by NKG2D-ULBP2 interaction. The results extends our understanding of gemcitabine in the treatment of pancreatic cancer from cell proliferation inhibition to immune regulation.

## INTRODUCTION

Pancreatic cancer has become the forth leading cause of cancer-related death worldwide, and is one of the most common malignant tumors [1, 2]. Radical surgery remains the only option to cure pancreatic cancer, but few patients are diagnosed when surgical resection is feasible [3]. That is why the prognosis is poor, with similar mortality and incidence [4]. The average survival period after prognosis is no more than six months, whereas the overall 5-year survival rate is less than 5%[5]. Therefore, it is urgent to develop novel therapeutic approaches to treat pancreatic cancer.

Gemcitabine is the only chemotherapy drug that has been demonstrated to show benefit in patients with pancreatic cancer [6]. Gemcitabine alone or in combination with other chemotherapy drug or radiation treatment may prolong survival of pancreatic cancer patients. It is reported to show an broad-spectrum anti-tumor effect for most solid tumors by destroying cell replication as a nucleotide analog [7]. However, little is reported about its regulation on cancer immunity. Gemcitabine may increase memory T cells and induce naïve T cell activation, and may enhance antitumor immunity induced by tumor vaccine [8, 9]. To expand the application of gemcitabine in treatment of pancreatic cancer, its immunological impact needs to be evaluated.

ULBP2, one of UL16-binding protein family, is a cell surface glycoprotein and functions as a stress-induced ligand for NKG2D receptor [10]. Various NKG2D ligands are shown to be upregulated by a range of primary tumors, including lung, kidney, prostate, breast and colon cancers [11–14]. Immune response induced by ULBP2-NKG2D may play an important role in the eradiation of tumors by T and/or NK cells.

In the present study, we investigated the correlation between the sULBP2 expression and gemcitabine, and found gemcitabine inhibit sULBP2 shedding from cell surface of pancreatic cancer cell lines, which protect pancreatic cancer from NK cells cytotoxicity. Furtherly, ADAM10 knockdown experiments demonstrated the essential roles of ADAM10 protease in the shedding of ULBP2. Gemcitabine showed anti-cancer effect by down-regulating NK cells function via inhibition of ADAM10 expression and shedding of sULBP2, which broadens our previous understanding of gemcitabine in the treatment of pancreatic cancer.

## RESULTS

### Gemcitabine inhibits shedding of ULBP2 in PANC-1 and MIA PACA-2 cells

**Figure 1:**
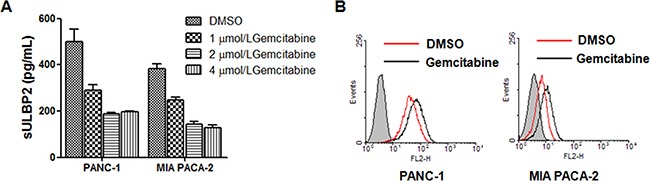
Gemcitabine inhibits shedding of ULBP2 in PANC-1 and MIA PACA-2 cells **A.** PANC-1 cells and MIA PACA-2 cells were treated with different concentrations of gemcitabine or vehicle (DMSO) for 24 h, and ULBP2 concentration was determinated by ELISA. **B.** Cells were treated with 2 μmol/l gemcitabine or vehicle (DMSO) for 24 h and membrane-bound ULBP2 expression was evaluated by flow cytometry.

We cultured 2 pancreatic cancer cell lines, PANC-1 and MIA PACA-2 cells and analyzed culture supernatants from the two cell lines. The level of sULBP2 decreased after gemcitabine was added to the culture medium of PANC-1 and MIA PACA-2 cells (Figure 1a). Gemcitabine was found to inhibit shedding of ULBP2 at concentrations of ≤ 2 μmol/L. Based on this finding, gemcitabine with concentrations of 2 μmol/l was used to in the next experiments. FACS analysis showed ULBP2 was expressed on the cell surface on PANC-1 and MIA PACA-2 cells in the membrane form, and gemcitabine upregulated ULBP2 surface expression (Figure 1b). Treatment with gemcitabine was observed to have markedly augmented membrane-bound ULBP2 expression and significantly decreased sULBP2 in PANC-1 cells and MIA PACA-2 cells.

### Gemcitabine enhances NK cells cytotoxicity to PANC-1 and MIA PACA-2 cells via ULBP2

**Figure 2:**
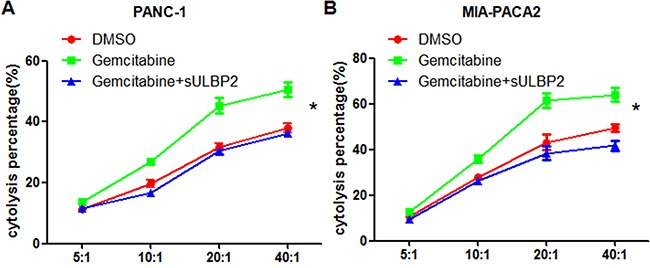
Gemcitabine enhances NK cells cytotoxicity to PANC-1 and MIA PACA-2 cells via sULBP2 The CCK-8 assay was used to determine the cytotoxicity of NK92 cells to PANC-1 cells **A.** and MIA-PACA2 cells **B.** Cells were treated with 2 μmol/l of gemcitabine (green) or vehicle (DMSO, red) for 4 h, and recombinant sULBP2 protein was added(blue).

As a ligand of nature immune activating receptor NKG2D, ULBP2-NKG2D interaction may promote tumors immune evasion. We cultured NK92 cell lines and evaluated the cytotoxicity of NK92 cells to PANC-1 or MIA PACA-2 cells using the CCK-8 assay. We co-cultured NK92 cells and PANC-1 or MIA PACA-2 cells, with or without gemcitabine. Treatment with gemcitabine was shown to enhance NK cytotoxicity to PANC-1 and MIA PACA-2 cells, whereas sULBP2 protein decreased NK cytotocity to PANC-1 cells or MIA PACA-2 cells remarkably (Figure 2a, 2b). The results demonstrated gemcitabine may have effect on NK cells function to pancreatic cancer cells via NKG2D-ULBP2 pathway.

### Gemcitabine inhibits ULBP2 shedding via suppressing ADAM10 expression

**Figure 3:**
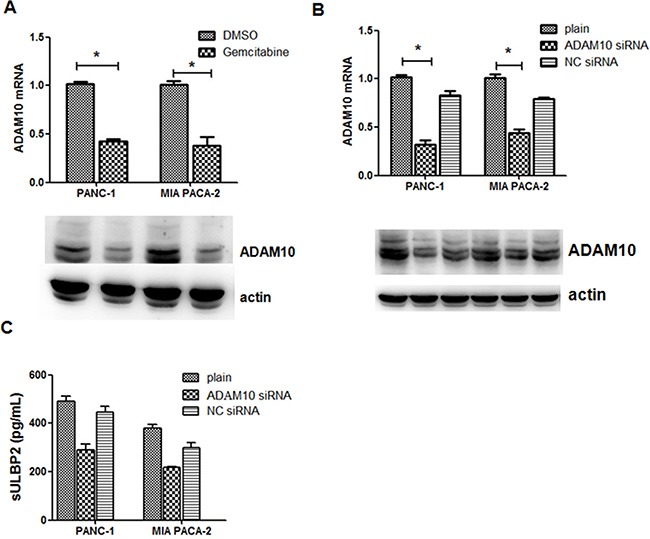
Gemcitabine-mediated shedding of ULBP2 is ADAM10-dependent **A.** ADAM10 expression of PANC-1 and MIA PACA-2 cells was determined when 2 μmol/l gemcitabine was added into cell culture. **B.** Cells were transfected with ADAM10 siRNA or control siRNA for 48 h and the expression of mRNA and protein of ADAM10 was evaluated by Q-PCR and western blot. **C.** sULBP2 in the culture supernatant was evaluated by ELISA. *P<0.05.

ADAM10 (a disintegrin and metalloproteinase 10) is reported to be responsible for the shedding of NKG2D ligands from the surface of various cell types via the proteolytic cleavage and release of the ectodomains of NKG2D ligands. To verify whether gemcitabine inhibits ULBP2 shedding through the suppression of ADAM10, we cultured PANC-1 cells and MIA PACA-2 cells with or without gemcitabine. Realtime PCR and western blot results showed that gemcitabine treatment downregulate ADAM10 expression in PANC-1 cells and MIA PACA-2 cells (Figure 3a). Then PANC-1 cells or MIA PACA-2 cells were transfected with siRNA against human ADAM10 or control siRNA. The downregulation of ADAM10 transcripts and ADAM10 protein was monitored by real-time RT-PCR and by western blotting, respectively (Figure 3b). No difference was noted in proliferation between the control and ADAM10 knockdown cells (data not shown). Knockdown of ADAM10 for PANC-1 cells and MIA PACA-1 cells resulted in 40.95% and 42.7% reduction of sULBP2 levels in the culture medium, respectively (Figure 3c). Taken together, these results suggest that gemcitabine inhibits ULBP2 shedding in PANC-1 cells and MIA PACA-1 cells by downregulating the expression of ADAM10.

### sULBP2 level is correlated with poor prognosis and ADAM10 expression

**Figure 4:**
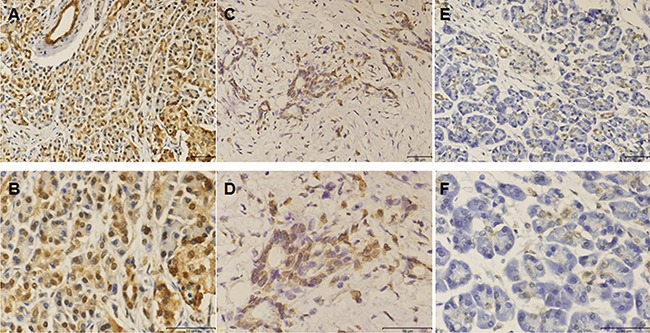
Immunohistochemical staining for ADAM10 The ADAM10 were principally localized in cytoplasm of tumor cells with varying staining intensity. **A.** High expression of ADAM10 (200x). **B.** High expression of ADAM10 (400x). **C.** Low expression of ADAM10 (200x). **D.** Low expression of ADAM10 (400x). **E.** Negative ADAM10 expression (200x). **F.** Partial enlargement of ADAM10 staining with the magnifying power of 400x.

**Figure 5:**
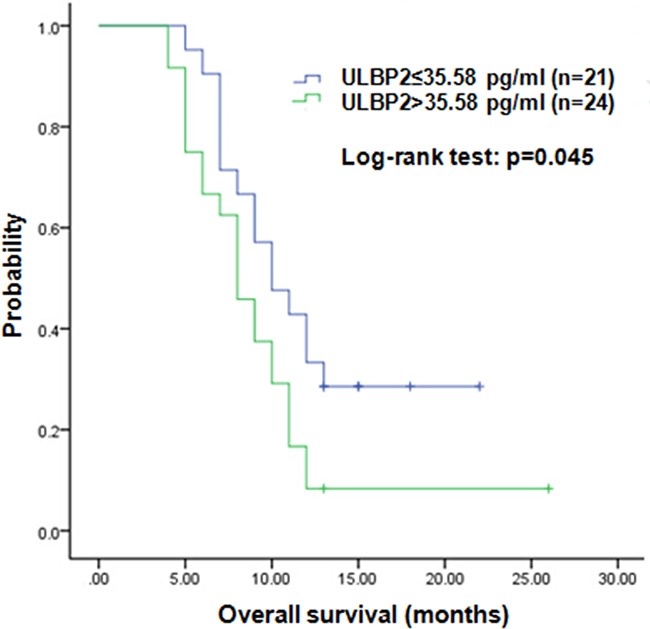
Kaplan-Meier analysis of overall survival sULBP2 low was defined as sULBP2 ≤ 35.58 pg/ml (median). sULBP2 high was defined as sULBP2 > 35.58 pg/ml. The P-value was determined using the log-rank test.

We next investigated serum levels of ULBP2 by ELISA assay in 45 PDAC patients (Supplementary Table S1) and 45 healthy individuals, and the sULBP2 levels of PDAC patients were significantly higher (p<0.001) than in healthy controls (data not shown). Based on ROC analysis of PDAC patients and healthy controls, the cut-off value of 16.11 pg/ml was used to divide the serum sample into groups that were negative or positive for sULBP2 (Supplementary Figure S1). The expression of ADAM10 was determined using immunohistochemical analysis, which showed that ADAM10 staining was mainly located in the cytoplasm of tumor cells with varying staining intensity (Figure 4). The clinical and pathological characteristics of the pancreatic cancer patients are presented in Table 1. A significant difference was observed in the serum ULBP2 levels with regard to the CA199 levels (p=0.013),lymph node metastasis (p=0.009) and overall survival(p=0.045)(Figure 5). There was no significant correlation between the ADAM10 expression with age, gender, tumor size, perineural invasion, or lymph node metastasis (P>0.05, respectively). Serum ULBP2 was found to be positively correlate with ADAM10 expression. The results indirectly confirmed that the effect of gemcitabine on pancreatic cancer may be related to ADAM10 and ULBP2.

**Table 1:** Correlation between ULBP2 and ADAM10 expression and clinicopathological characteristics

	n	ADAM10 staining		p	Serum ULBP2 (pg/ml)		p
		−/+	++/+++		<16.11	≥16.11	
All	45	6	39		4	41	
*Gender*							
female	16	0	16	0.171	0	16	0.261
male	29	6	23		4	25	
*Age*							
<60	19	2	17	0.141	0	19	0.238
≥60	26	4	22		4	22	
*CA19-9*							
<37U/ml	10	0	10	0.138	0	10	0.013
≥37U/ml	35	6	29		4	31	
*Tumor size (cm)*							
≤2	5	1	4	0.214	1	4	0.184
>2	40	5	35		3	37	
*Tumor location*							
Head	34	4	30	0.024	2	32	0.100
Body/tail	11	2	9		2	9	
*Histological grade*							
Well-mod	25	4	21	0.099	2	23	0.092
poor	20	2	18		2	18	
TNM stage							
I-II	34	5	29	0.078	3	31	0.059
III-IV	11	1	10		1	10	
Perineural invasion							
present	20	4	16	0.079	3	17	0.208
absent	25	2	23		1	24	
LNM							
present	17	1	16	0.098	1	16	0.009
absent	28	5	23		3	25	

## DISCUSSION

Gemcitabine is the standard chemotherapy regimen for the treatment of advanced pancreatic cancer [15]. It is a nucleoside analogue, which exerts its anti-tumor effect through a variety of mechanisms, mainly via inhibition of DNA replication and mask of DNA chain termination [16]. Therefore, it prevents the DNA synthesis and DNA repair, causes the cell to enter apoptotic pathway. However, its role in immune response have not be fully documented. Our results demonstrated that gemcitabine inhibit ULBP2 ectodomain shedding through down-regulation of ADAM10 expression in PANC-1 and MIA PACA-2 cell lines. Decrease of sULBP2 and increase of membrane-bound ULBP2 thus promote NK cells activation and may improve the antitumor effect against pancreatic cancer, which will be further confirmed by studies on tissues of pancreatic cancer patients.

In the present study, the level of serum ULBP2 was examined in 45 PDAC patients using ELISA. sULBP2 was found to be over-expressed in sera of pancreatic cancer patient compared with healthy individuals. Moreover, a significant difference was noted in the serum ULBP2 level with regard to the CA199 levels and lymph node metastasis. Serum levels of ULBP2 in pancreatic cancer patients were also found to correlate significantly with shorter overall survival and poor prognosis. ULBP2 shedding is thought to be a principal mechanism by which tumor cells escape from NKG2D-mediated immune surveillance in pancreatic cancer. Therefore, ULBP2 is hypothesized to be associated with the malignant transformation of pancreatic cancer.

ADAM10 is one of the the ADAM family of disintegrin proteinasesm, and mediates proteolytic release of ectodomains of transmembrane proteins from the cell surface, including cytokines, growth factors and cell adhesion molecules [17, 18]. The present study demonstrated that ADAM10 expression is significantly lower in gemcitabine treated pancreatic cell lines. Meanwhile, serum levels of ULBP2 was positively correlated with ADAM10 expression shown by immunohistochemistry, suggesting that gemcitabine may exerts its anti-tumor effect through inhibition of ADAM10 mediated ULBP2 shedding and corresponding enhanced NKG2D-mediated tumor elimination.

In this study, the siRNA mediated ADAM10 knockdown was found to decrease the release of sULBP2, which proved the essential role for the cleavage of sULBP2. The observations suggest that ADAM10 may serve as a potential therapeutic target for treatment of pancreatic cancer patients who are not sensitive to gemcitabine.

In conclusion, gemcitabine has been demonstrated not only to inhibit cell proliferation, but also to enhance the immune response of pancreatic cancer cells by enhancing the activity of NK cells. The present study sheds light on previously unrecognized effects of gemcitabine on tumor immune response, modulating ADAM10 and ULBP2 shedding, thus suggesting its use in chemoimmunotherapy against human pancreatic cancer.

## MATERIALS AND METHODS

### Subjects and samples

Forty-five patients with pancreatic cancer were enrolled between January 2013 and Dec 2015. The patients were surgically treated in the Affiliated Provincial Hospital of Anhui Medical University(Hefei, China). The samples of cancer tissue were obtained during surgery, and then fixed in 10% formalin solution and embedded in paraffin. The diagnosis of the samples was confirmed histopathologically. Informed consent was obtained from each patient, and the study protocol was approved by the Research Ethics Committee of Anhui Provincial Hospital. Follow-up data were collected from all 45 patients, and the mean follow-up period in the present investigation was 9.8 (range 4–26) months. Overall survival (OS) time was calculated from the date of surgery to the date of death or last observation.

### Cell lines

The PANC-1, MIA PaCa-2 pancreatic cancer cell lines and NK92 cell lines were obtained from Shanghai cell bank (Shanghai, China). PANC-1 cells were maintained in DMEM (GIBCO) with 10% heat inactivated fetal bovine serum(FBS), 50 units/mL penicillin, and 50 units/mL streptomycin. MIA PaCa-2 cells were cultured in DMEM supplemented with 10% FBS, 2.5% horse serum, 1% sodium pyruvate 100 mM solution (Invitrogen), 50 units/mL penicillin, and 50 units/mL streptomycin. NK92 cells were maintained in α-MEM (GIBCO) with 10% FBS, 100 U/mL recombinant human IL-2 (rhIL-2; PeproTech), 50 units/mL penicillin, and 50 units/mL streptomycin. All cells were cultured in humidified air with 5% CO_2_ at 37°C.

### Quantitative real-time PCR

Total RNA samples was extracted by using Trizol according to the manufacturer's protocol. Quantitative PCR was performed using SYBR Premix Ex Taq (Takara). The primer sequences used were as follows: ADAM10 forward, 5'- TTGGGAAGATGGTAGCTTGG -3'; reverse, 5'- CACATATTCCTCCAGAGCTTCC-3'; GAPDH mRNA served as internal control.

cDNA was amplified (40 cycles, 95°C for 10 sec, 60°C for 30 sec, 72 for30 sec) with an ABI StepOnePlus according to the manufacturer's instructions. A melting curve analysis was performed to monitor PCR-product purity, and relative gene expression data were analyzed using the 2-ΔΔ Ct method.

### Western blot

PANC-1 cells and MIA PACA-2 cells were lysed with lysis buffer (Beyotime Institute of Biotechnology, China), and protein concentration was subsequently estimated by the bicinchoninic acid protein assay. Then, equal amounts of protein samples were separated on 10% sodium dodecyl sulfate polyacrylamide gel electrophoresis gels and transferred onto polyvinylidene difluoride membranes (Millipore, USA). After being blocked with 5% nonfat milk, membranes were incubated overnight at 4 °C with rabbit primary antibodies against human ADAM10 (Boster, China) and human actin (Zhongshan Golden Bridge Biotechnology, China). After being washed with Tris-buffered saline/0.1% Tween three times, membranes were incubated with secondary antibodies for 1 hours at room temperature. Immunoreactive bands were visualized using the enhanced chemiluminescence (ECL; Pierce,USA) kit. The specific protein bands were captured and visualized using FUSION FX5 (Vilber Lourmat).

### ELISA assay

Blood samples were procured from the pancreatic cancer patients with informed consent according to the institutional guidelines. Serum samples were centrifuged at 1500 g for 10 min and the sera were stored at −80 °C until usage. Supernatants of the cell lines were harvested at 72hr. For the determination of ULBP2 levels, commercial DuoSet ELISA kits (R&D Systems) was used, and assays were performed following the manufacturer's instructions. The data were analyzed with Origin 8.0 software.

### siRNA-mediated knockdown

The small interfering RNA (siRNA) method was used to knockdown ADAM10. Chemically synthesized ADAM10 siRNA and negative control siRNA were purchased from Genechem (Shanghai, China). Lipofectamine 2000 (Invitrogen) was used for transfection following the manufacture's instructions. The siRNAs used were: ADAM10, 5′-AACCCAGCTGTCACCCTCGAA-3′; negative control, 5′-TTCGAGGGTGACAGCTGGGTT-3′.

### Flow cytometric analysis

For the detection of membrane-bound ULBP2, cells were incubated with phycoerythrin (PE)-mouse anti ULBP2 antibody (FAB1298P, R&D systems) and then subjected to flow cytometry. Flow cytometry was performed using a FACSCalibur, and data were analyzed using WinMDI2.9 software.

### CCK-8 assay

The CCK-8 (cell counting kit-8) assay was used to determine the cytotoxicity of NK92 cells. PANC-1 or MIA PACA-2 cells were used as target cells. Target cells were seeded in 96-well plates, with 1*104 cells in each well, with or without gemcitabine. NK92 cells were incubated with target cells at effector/target ratios of 1:1, 1:5, 1:10 or 1:25 for 4 hr. A 20 μL aliquot of enhanced CCK8 solution (5 mg/ml in PBS) was then added to each well. Following 1 hr of incubation, absorbance was measured on an ELISA reader at a test wavelength of 450 nm. The percentage of cytolysis was calculated as [1-(Effect with Target cells)/Target]×100.

### Immunohistochemistry

Serial tissue sections (4 μ m thick) were deparaffinized with xylene, rehydrated, and subjected to microwave antigen retrieval in citrate buffer (pH 6.0) for 20 minutes. Endogenous peroxidase activity was quenched by 3% hydrogen peroxide for 10 minutes. Afterward, the sections were incubated at 4^°^ C overnight with monoclonal antibody (anti-ADAM10; Abcam) in a humidified chamber. After being washed, the sections were incubated for 30 minutes with horseradish peroxidase-conjugated secondary antibody (Zhongshan Golden Bridge Biotechnology). Immunoreactivity was visualized with chromogen 3,3'diaminobenzidine. Finally, all slides were counterstained with ematoxylin, dehydrated, and mounted. Tumor expression of ADAM10 was semiquantitatively evaluated using a previously reported method, and the percentage of staining cell scores (0, no staining; 1, 10%; 2, 10%–30%; 3, >30%) and the staining intensity scores (0, negative; 1, weak; 2, moderate; 3, strong) were summed. The final scores were ranged as following: total score < 3 was defined as negative (−), 3-6 as“+”, 6-9 as“++'', and 9-12 as “+++''. All sections were assessed by two pathologists who were blinded to clinical data.

### Statistical analysis

Data were expressed as the mean ± SD. The means between groups were analyzed using the Mann-Whitney U test. All statistical analyses were performed using SPSS 16.00 statistical analysis software (SPSS Inc). Differences were considered statistically significant if p< 0.05.

## SUPPLEMENTARY FIGURE AND TABLE




