# Supplementary material for: Gemcitabine inhibits immune escape of pancreatic cancer by down regulating the soluble ULBP2 protein
Source: Oncotarget. 2016 Sep 1;7(43):70092–9. doi: 10.18632/oncotarget.11780 (PMC5342537; doi:10.18632/oncotarget.11780)
Supplement: Supplementary file 1 [file oncotarget-07-70092-s001.pdf]

Gemcitabine inhibits immune escape of pancreatic cancer by down regulating the soluble ULBP2 protein

SUPPLEMENTARY FIGURE AND TABLE

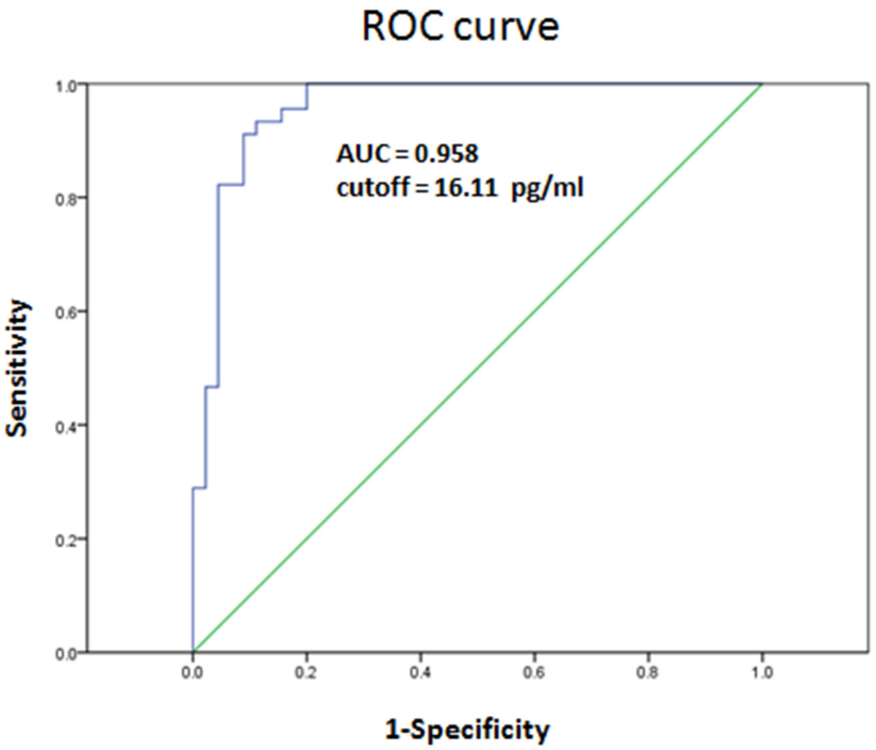

Supplementary Figure S1: ROC analysis of serum sULBP2 concentration in 45 PDAC patients and 45 healthy individuals. The sULBP2 levels of PDAC patients were significantly higher ($p<0.001$) than in healthy controls. Based on ROC analysis of PDAC patients and healthy controls, the cut-off value of 16.11 pg/ml was used to divide the serum sample into groups that were negative or positive for sULBP2.

Supplementary Table S1: Clinical characters of PDAC patients. The largest size of multiple tumors was shown in tumor size.

See Supplementary File 1
